# *Atg7*-dependent canonical autophagy regulates the degradation of aquaporin 2 in prolonged hypokalemia

**DOI:** 10.1038/s41598-019-39702-4

**Published:** 2019-02-28

**Authors:** Wan-Young Kim, Sun Ah Nam, Arum Choi, Yu-Mi Kim, Sang Hee Park, Hong Lim Kim, Hyang Kim, Ki-Hwan Han, Chul Woo Yang, Myung-Shik Lee, Yong Kyun Kim, Jin Kim

**Affiliations:** 1 Department of Anatomy and Cell Death Disease Research Center, College of Medicine, The Catholic University of Korea, Seoul, Korea; 2 Institute of Clinical Medicine Research of Bucheon St. Mary’s Hospital, Bucheon, Korea; 3 Integrative Research Support Center, College of Medicine, The Catholic University of Korea, Seoul, Korea; 4 Division of Nephrology, Kangbuk Samsung Hospital, Sungkyunkwan University, School of Medicine, Seoul, Korea; 5 Department of Anatomy, Ewha Womans University School of Medicine, Seoul, Korea; 6 Department of Internal Medicine, College of Medicine, The Catholic University of Korea, Seoul, Korea; 7 Severance Biomedical Science Institute, College of Medicine, Yonsei University, Seoul, Korea

## Abstract

Prolonged hypokalemia induces a decrease of urinary concentrating ability via down-regulation of aquaporin 2 (AQP2); however, the precise mechanisms remain unknown. To investigate the role of autophagy in the degradation of AQP2, we generated the principal cell-specific *Atg7* deletion (*Atg7*^Δpc^) mice. In hypokalemic *Atg7-*floxed (*Atg7*^f/f^) mice, huge irregular shaped LC3-positive autophagic vacuoles accumulated mainly in inner medullary collecting duct (IMCD) cells. Total- and pS261-AQP2 were redistributed from apical and subapical domains into these vacuoles, which were not co-localized with RAB9. However, in the IMCD cells of hypokalemic *Atg7*^Δpc^ mice, these canonical autophagic vacuoles were markedly reduced, whereas numerous small regular shaped LC3-negative/RAB9-positive non-canonical autophagic vacuoles were observed along with diffusely distributed total- and pS261-AQP2 in the cytoplasm. The immunoreactivity of pS256-AQP2 in the apical membrane of IMCD cells was markedly decreased, and no redistribution was observed in both hypokalemic *Atg7*^f/f^ and *Atg7*^Δpc^ mice. These findings suggest that AQP2 down regulation in hypokalemia was induced by reduced phosphorylation of AQP2, resulting in a reduction of apical plasma labeling of pS256-AQP2 and degradation of total- and pS261-AQP2 via an LC3/ATG7-dependent canonical autophagy pathway.

## Introduction

Prolonged hypokalemia induces a vasopressin-resistant decrease of urinary concentration and polyuria, which is caused by down-regulation of the expression of aquaporin 2 (AQP2)^1–4^. AQP2 abundance is determined by the balance between its production by translation and its removal by degradation or exosomal excretion^5,6^. Although AQP2 degradation occurs via lysosomes or proteasomes, the precise mechanisms underlying this phenomenon remain unknown. Recently, it has been suggested that arginine vasopressin-mediated phosphorylation can regulate AQP2 abundance^7^. Furthermore, the complexity of AQP2 regulation was revealed by the discovery that AQP2 can be phosphorylated at several sites. To date, 5 potential phosphorylation sites on the AQP2 C-terminus have been determined: Thr244, Ser256, Ser261, Ser264, and Ser269^8–11^. Phosphorylation of AQP2 at Ser256 (pS256-AQP2) and Ser261 (pS261-AQP2) may inversely regulate the endocytosis and exocytosis of AQP2^8,12–15^. In particular, pS256-AQP2 is necessary for the regulated membrane accumulation of AQP2, which leads to increased water reabsorption and urinary concentration^12,16–19^. In contrast, pS261-AQP2 is proposed to stabilize AQP2 ubiquitination and intracellular localization and counterbalances pS256-AQP2^8,12,19^.

Prolonged hypokalemia is a consequence of a common imbalance in potassium (K^+^) levels that can cause defects in urinary concentration ability, i.e., nephrogenic diabetes insipidus (NDI) in humans and experimental animals, including mice^2,3,20–22^. The collecting duct (CD) is the main nephron site where morphological alterations occur during K^+^ deficiency. Among the various morphological changes observed, the most remarkable is the accumulation of cytoplasmic droplets in collecting duct cells^21,23,24^ that are believed to represent lysosomal structures^20,25^ and are labeled by AQP2^2^. However, even though these droplets are formed in a classical rodent model of hypokalemia induced by ingestion of K^+^ free diet for a period of 2 weeks, it is not certain whether they comprise autophagic vacuoles or are involved in AQP2 degradation.

Autophagy is a self-digesting process that is essential for the survival of eukaryotic cells, whereby unnecessary materials and dysfunctional organelles are sequestered and delivered into lysosomes for degradation^26–28^. Notably, this process represents a catabolic pathway utilized to maintain a balance among the synthesis, degradation, and recycling of cellular components, thereby playing a role in homeostasis^27,29^. However, until recently, the role of autophagy in kidney physiology has remained a largely understudied topic. The discovery of autophagy-related genes (*ATGs*) has greatly enhanced our understanding of the mechanisms of the autophagic pathway^30–32^. Formation of the autophagosome requires two unique ubiquitin-like protein conjugation systems: the ATG5-ATG12 pathway, and the LC3 pathway^33^. ATG7 acts as a catalyst in both conjugation systems and is therefore essential for autophagy^34,35^. This ATG7/ATG5/LC3-II-dependent pathway is called canonical (conventional) autophagy. In addition, an ATG7/ATG5/LC3-II-independent pathway termed noncanonical (alternative) autophagy has also recently been described^36–41^.

In this study, we examine the role of canonical or non-canonical autophagy in the degradation of AQP2, with particular focus on pS256- and pS261-AQP2 in induced prolonged hypokalemia. To this end, we generated conditional knockout mice in which ATG7 was genetically ablated specifically in AQP2-positive principal cells of the collecting duct. We restricted our observations to the IMCD cells that are main site of hypokalemia-associated morphological changes including the accumulation of cytoplasmic droplets.

## Results

### Principal cell-specific Atg7 deficiency mice exhibit enhanced polyuria and urinary concentration defects during hypokalemia

To address the role of autophagy in renal AQP2 homeostasis, we generated principal cell-specific Atg7-knockout (*Atg7*^Δpc^) mice by breeding *Atg7-*floxed mice (*Atg7*^f/f^) (kindly provided from Dr. Komatsu, Japan) with AQP2-Cre mice (Jackson Laboratories).

To study hypokalemia, mice were fed either normal diet or K^+^-free diet for 2 weeks. While on a normal diet, the serum K^+^ concentration was not significantly altered between *Atg7*^f/f^ and *Atg7*^Δpc^ mice. Following 2 weeks of dietary K^+^ depletion, serum K^+^ and urinary K^+^excretion decreased on the K^+^-deficient diet in both *Atg7*^f/f^ and *Atg7*^Δpc^ mice compared with control groups. Thus, 2 weeks of K^+^-free diet induced hypokalemia in both *Atg7*^f/f^ and *Atg7*^Δpc^ mice.

After 2 weeks on reduced K^+^ diet, a significant increase in urine volume and a significant reduction in urine osmolality were observed in both hypokalemic *Atg7*^f/f^ and *Atg7*^Δpc^ mice and these changes in the *Atg7*^Δpc^ mice subsequently became pronounced compared with *Atg7*^f/f^ mice. Even in the basal condition a significant increase in urine volume and a significant reduction in urine osmolality were observed in the *Atg7*^Δpc^ mice compared to the *Atg7*^f/f^ mice. These findings indicate that polyuria and enhanced urinary concentration are induced in K^+^ depleted *Atg7*^f/f^ mice and that these changes in *Atg7*^Δpc^ mice became pronounced.

Renal hypertrophy together with a urinary concentrating defect is normally considered hallmark of potassium depletion induced by restricting potassium intake^1^, therefore we analyzed kidney weights normalized for body weight for evidence of renal hypertrophy. Setting the *Atg7*^f/f^ control group as 100%, a significant increase in kidney weight normalized for body weight was observed in *Atg7*^Δpc^ mice maintained on a low K^+^ diet compared with knockout mice on a normal K^+^ diet, revealing a marked hypertrophy. Serum BUN levels were significantly decreased in *Atg7*^Δpc^ mice maintained on a low K^+^ diet compared with those on a normal K^+^ diet, whereas no significant changes were not observed between low K^+^ diet group and normal K^+^ diet group in the *Atg7*^f/f^ control mice. It may presumed that the markedly increased urine volume in *Atg7*^Δpc^ mice maintained on a low K^+^ diet cause dehydration and subsequently contributed prerenal acute kidney injury. These chemical data were summarized at Supplementary Table S1.

### Hypokalemia induces autophagy in the IMCD cells of *Atg7*^*f/f*^ mice

To determine whether the cytoplasmic droplets in the CD induced by K^+^ depletion represent autophagic vacuoles, we monitored the kidney after 2 weeks of K^+^-free diet using immunoblotting and immunohistochemistry for LC3, as well as by ultrastructural analysis.

**Figure 1:**
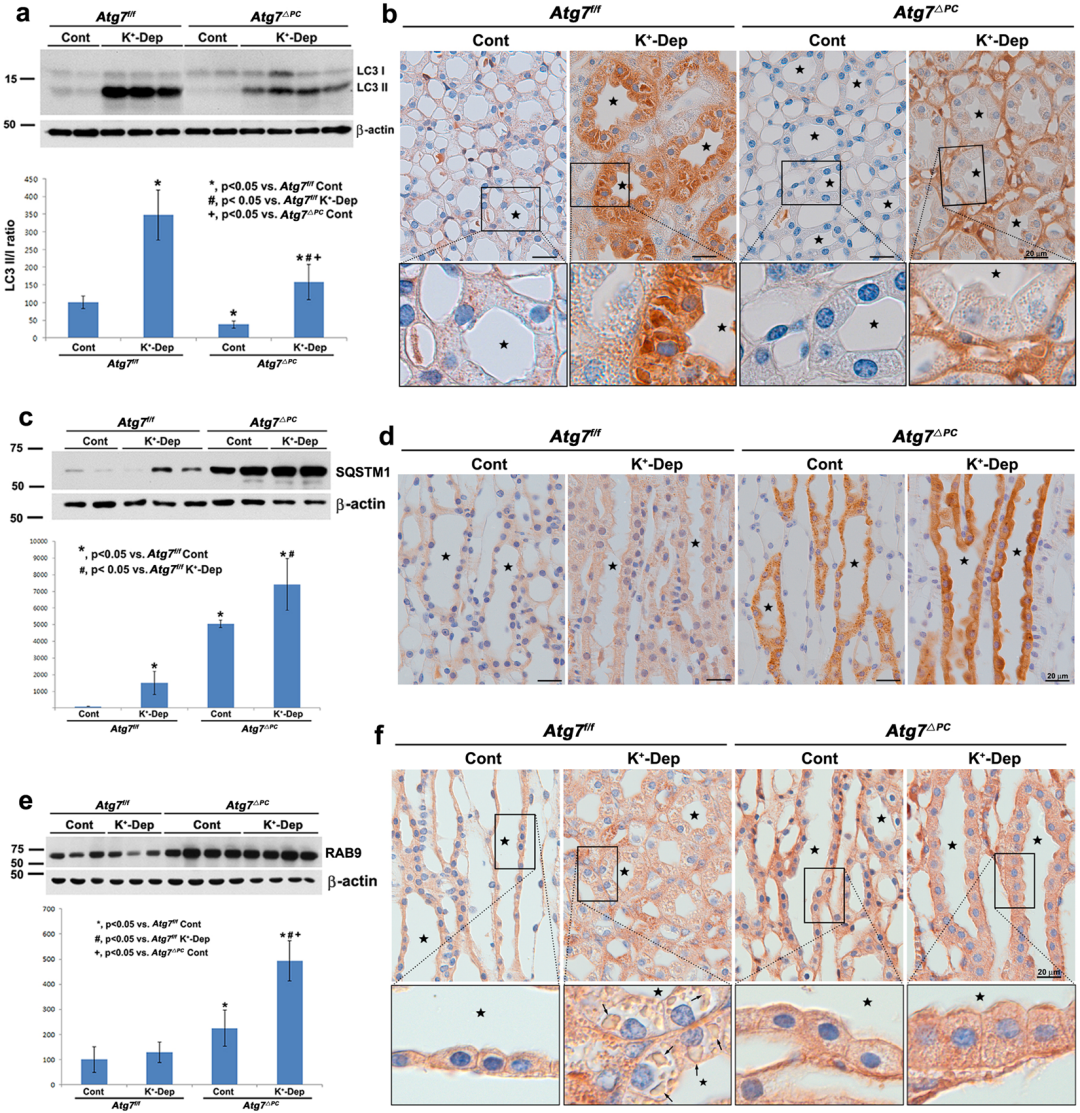
Light micrographs of the inner medulla of control (Cont) and K^+^-depleted (K^+^-Dep) *Atg7*^f/f^ and *Atg7*^Δpc^ mouse kidneys illustrating immunoblotting (**a**,**c**,**e**) and immunolabeling (**b**,**d**,**f**) of LC3 (**a**,**b**), SQSTM1 (**c**,**d**) and RAB9 (**e**,**f**). (**a**) The degree of increase in the LC3 II/I ratio is significantly decreased in K^+^-depleted *Atg7*^Δpc^ compare to K^+^-depleted *Atg7*^f/f^ mice. (**b**) In the images, even in the control groups, immunoreactivity for LC3 in the inner medullary collecting duct (IMCD, stars) cells are decreased in *Atg7*^Δpc^ mice compare to *Atg7*^f/f^ mice. After K^+^ depletion, immunoreactivity for LC3 in dramatically increased in the IMCD cells of *Atg7*^f/f^ mice. However, immunoreactivity for LC3 is restrictively decreased in the IMCD cells of K^+^-depleted *Atg7*^Δpc^ mice. Note that immunoreactivity for LC3 in other structures including interstitial cells and thin limb cells of Henle’s loop of renal papilla in K^+^-depleted *Atg7*^Δpc^ mice is stronger in K^+^-depleted *Atg7*^f/f^ mice, but relatively weaker than that of IMCD cells in K^+^-depleted *Atg7*^f/f^ mice. (**c**) The protein level of SQSTM1 is significantly increased in *Atg7*^Δpc^ compare to *Atg7*^f/f^ mice. (**d**) In the images, strong immunoreactivity for SQSTM1 is observed restrictively in the IMCD cells of *Atg7*^Δpc^ mice, which change is pronounced after K^+^ depletion. (**e**) Note the prominent increase of RAB9 in K^+^-Dep *Atg7*^Δpc^ mice. (**f**) Immunoreactivity for RAB9 is not observed in the autophagic vacuoles (arrows) of K^+^-Dep *Atg7*^f/f^ mice. However, it is expressed in small autophagic vacuoles of K^+^-Dep *Atg7*^Δpc^ mice. Boxes in (**b**,**f**) are higher magnification of the areas indicated by the rectangles in upper panels. Values represent the means ± SD.

Immunoblotting of whole of renal inner medulla proteins showed that the conversion of LC3-I to LC3-II was markedly increased in K^+^-depleted *Atg7*^f/f^ mice (Fig. 1a), whereas LC3 immunohistochemistry revealed small numbers of tiny LC3-positive droplets scattered throughout the cytoplasm in *Atg7*^f/f^ mice fed a normal K^+^ diet (Fig. 1b, Supplementary Fig. S1a1′). Following 2 weeks of dietary K^+^ depletion in *Atg7*^f/f^ mice, the most pronounced LC3-positive droplet accumulation occurred in AQP4-positive IMCD cells, wherein the LC3-positive droplets were large in size, irregular in shape, and often acquired gigantic proportions (Fig. 1b, Supplementary Fig. S1a2′). In contrast, although numerous cytoplasmic inclusions could be seen in the thin limb cells of Henle’s loop, the interstitial cells, and the endothelial cells of the papillary region, immunoreactivity for LC3 was only faintly observed in the majority of these inclusions (Supplementary Fig. S1a2′). In the cortex and the outer medulla, however, the LC3 II/I ratio was not significantly changed in K^+^-depleted *Atg7*^f/f^ mice and the LC3-positive droplet accumulation was relatively sparse in all structures including the cortical and outer medullary CDs. We confirmed same findings using GFP-LC3 transgenic mice (Supplementary Fig. S2). These findings indicate that hypokalemia induced autophagy restrictively in the renal papilla including in IMCD cells in *Atg7*^f/f^ mice.

### Non-canonical autophagy is activated in the IMCD cells of K^+^-depleted *Atg7*^Δpc^ mice

In K^+^-depleted *Atg7*^Δpc^, western blot analyses revealed that the degree of LC3 II/I ratio increase was less than that of K^+^-depleted *Atg7*^f/f^ mice (Fig. 1a). Even in the normal diet groups, the LC3 II/I ratio and the immunoreactivity for LC3 in *Atg7*^Δpc^ mice was decreased compare with those of Atg7^f/f^ mice (Fig. 1a,b). Notably, in K^+^-depleted *Atg7*^Δpc^ mice, LC3-positive droplets in the IMCD cells were markedly decreased compare with those in K^+^-depleted *Atg7*^f/f^ mice (Fig. 1b). However, numerous LC3-negative cytoplasmic small round droplets of regular size were observed in these IMCD cells (Fig. 1b). In addition, in K^+^-depleted *Atg7*^Δpc^ mice, although immunoreactivity for LC3-II increased in the interstitial cells, endothelial cells, and thin limb cells of Henle’s loop, a marked decrease of immunoreactivity for LC3-II was observed restrictively in the IMCD cells (Fig. 1b).

Furthermore, p62/sequestosome 1 (SQSTM1) was significantly increased only in the IMCD cells of *Atg7*^Δpc^ mice especially with K^+^-depletion for 2 weeks (Fig. 1c,d), suggesting that, in the renal papilla, autophagy was selectively inhibited in the IMCD cells of *Atg7*^Δpc^ mice. These findings suggest that the increased autophagic vacuoles in *Atg7*^Δpc^ mice after K^+^-depletion represent the autophagy generated in an Atg7-independent manner (ATG5/ATG7-independent non-canonical autophagy)^36,37^. To confirm these findings, we performed western blot analyses and immunohistochemistry for the RAB9, which is essential for membrane expansion and fusion in non-canonical autophagy^36^. The protein abundance of RAB9 was significantly increased in both control and K^+^-depleted *Atg7*^Δpc^ mice compared with *Atg7*^f/f^ mice (Fig. 1e,f). These findings indicated that ATG7-independent non-canonical autophagy was induced restrictively in the IMCD cells of *Atg7*^Δpc^ mice. Therefore, we restricted our subsequent ultrastructural observations to the IMCD cells, in which there was a marked alteration of LC3-positive autophagic vacuoles between *Atg7*^f/f^ and *Atg7*^Δpc^ mice.

### Ultrastructural characteristics of autophagic vacuoles induced by prolonged hypokalemia

**Figure 2:**
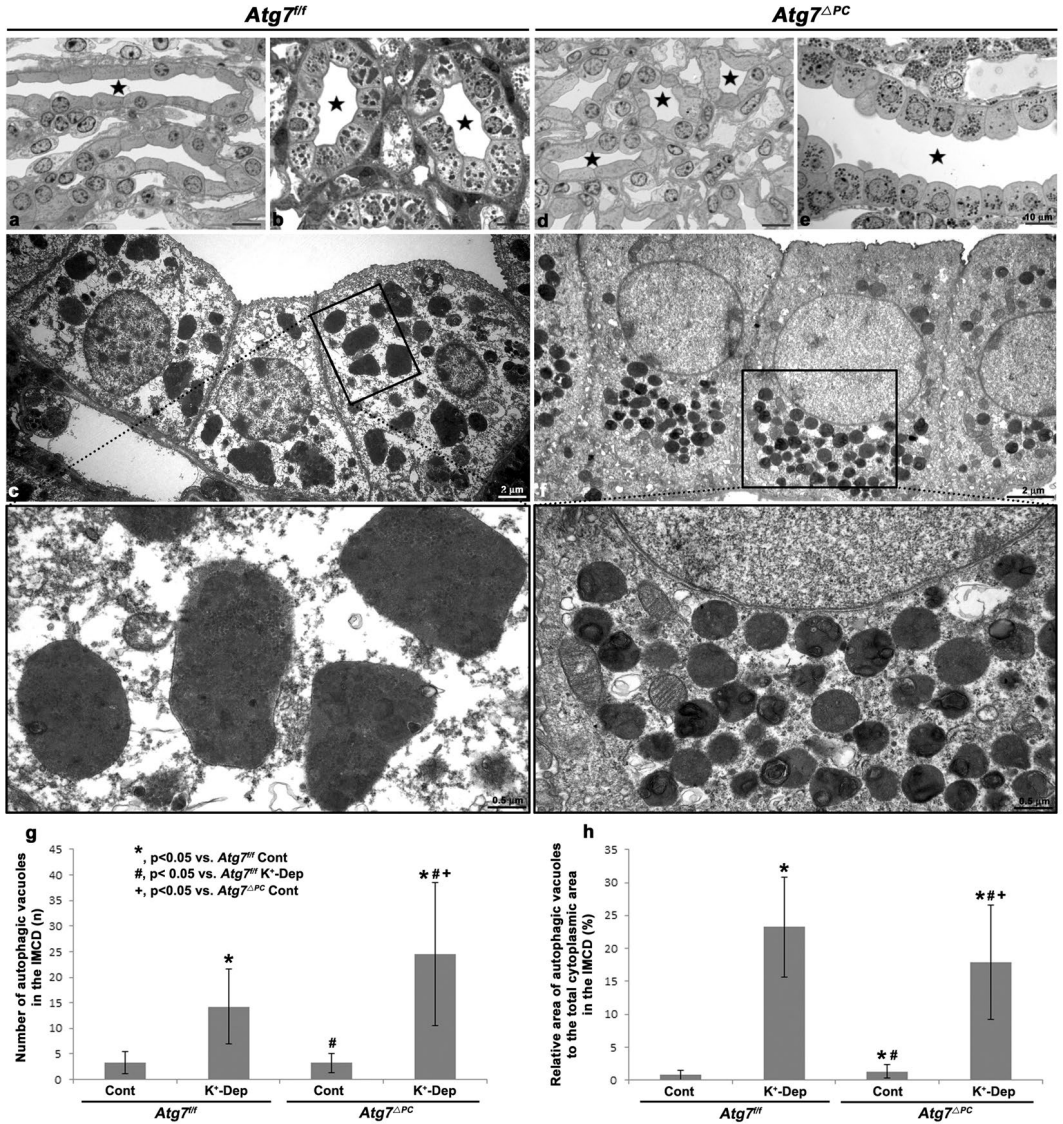
Light (**a**,**b**,**d**,**e**) and transmission electron (**c**,**f**) micrographs of the inner medulla of control (**a**,**d**) (Cont) and K^+^-depleted (**b**,**c**,**e**,**f**) (K^+^-Dep) *Atg7*^f/f^ (**a**–**c**) and *Atg7*^Δpc^ (**d**–**f**) mouse kidneys. Stars indicate the lumen of the inner medullary collecting duct (**a**,**b**,**d**,**e**). The non-canonical autolysosomes in K^+^-Dep *Atg7*^Δpc^ mice are much greater in number (**g**) and smaller in size than those of canonical autophagic vacuoles in K^+^-Dep *Atg7*^f/f^ mice. However, the relative area of autophagic vacuoles to the total cytoplasmic area is significantly decreased in K^+^-Dep *Atg7*^Δpc^ compared to *Atg7*^f/f^ mice (**h**).

Electron microscopy revealed that the IMCD cells of K^+^-depleted *Atg7*^f/f^ mice contained large irregular shaped autophagic vacuoles containing multilamellar bodies, small vesicles and granular inclusions (Fig. 2a–c). Notably, although no LC3-positive puncta were observed in the descending and ascending thin limb cells, the interstitial cells, and the endothelial cells of the papillary region of K^+^-depleted *Atg7*^f/f^ mice via light microscopic immunohistochemistry (Fig. 1b), we could observe many autophagic vacuoles in these structures (Supplementary Fig. S3).

Despite the disappearance of LC3-positive droplets in the IMCD cells of K^+^-depleted *Atg7*^Δpc^ mice under light microscopic immunohistochemistry (Fig. 1b), ultrastructural analysis revealed numerous small, uniformed autophagic vacuoles with electron-dense materials (Fig. 2d–f). Furthermore, whereas the number of autophagic vacuoles was increased, the fractional area of the autophagic vacuoles was significantly decreased in *Atg7*^Δpc^ mice compared with *Atg7*^f/f^ mice after K^+^-depletion (Fig. 2g,h). These autophagic vacuoles in the IMCD cells of K^+^-depleted *Atg7*^Δpc^ mice might comprise LC3-negative non-canonical autophagic vacuoles. In addition, in the cytoplasm of both control and K^+^-depleted *Atg7*^f/f^ mice, cell organelles including Golgi complex and vesicles were sparse but polysomes were relatively well developed (Supplementary Fig. S4a–c). In contrast, in both control and K^+^-depleted *Atg7*^Δpc^ mice, well developed Golgi complexes and small vesicles were observed in the IMCD cells (Supplementary Fig. S4d–f). Taken together, these data suggest that, in the IMCD cells of K^+^-depleted *Atg7*^f/f^ mice, LC3-positive canonical autophagy was induced. In contrast, LC3-negative non-canonical autophagy was induced in the IMCD cells of K^+^-depleted *Atg7*^Δpc^ mice.

### Alteration of distribution and amount of total-, pS261-, and pS256-AQP2

**Figure 3:**
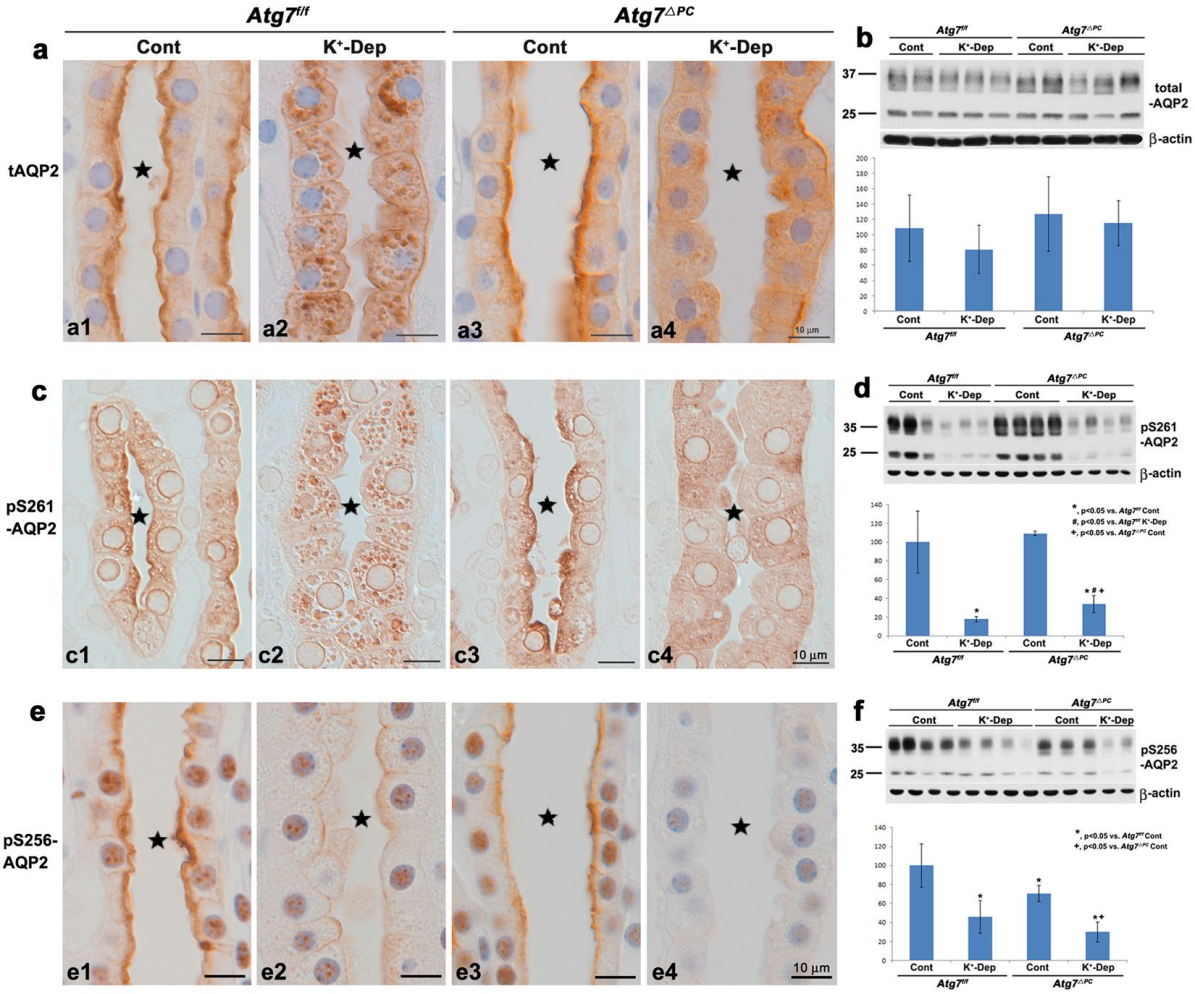
Light micrographs of the inner medulla of control (Cont) and K^+^-depleted (K^+^-Dep) *Atg7*^f/f^ and *Atg7*^Δpc^ mouse kidneys illustrating immunolabeling (**a**,**c**,**e**) and immunoblotting (**b**,**d**,**f**) of total AQP2 (tAQP2) (**a**,**b**), pS261-AQP2 (**c**,**d**) and pS256-AQP2 (**e**,**f**). (**a**) tAQP2 are immunolabeled in the apical and subapical domains of the inner medullary collecting duct (IMCD, stars) cells in both control *Atg7*^f/f^ and *Atg7*^Δpc^ mice. After K^+^ depletion, however, tAQP2 can be observed in intracellular vesicles and throughout the cytoplasm in K^+^-Dep *Atg7*^f/f^ and *Atg7*^Δpc^ mice, respectively. (**c**) Note those, after K^+^ depletion, changes of intracellular pattern of immunoreactivity of pS261-AQP2 are similar to those of tAQP2 as shown in (**a**). (**e**) It can be observed that apical pS256-AQP2 immunolabeling is markedly reduced in K^+^-Dep *Atg7*^f/f^ mice, and this change is pronounced in K^+^-Dep *Atg7*^Δpc^ mice. (**b**,**d**,**f**) The protein amount of pS261-AQP2 and pS256-AQP2 are significantly decreased after both K^+^-depletion compared with controls in both *Atg7*^f/f^ and *Atg7*^Δpc^ mice.

To investigate the effect of hypokalemia on the expression and distribution of AQP2, we performed western blot analyses and immunohistochemical staining for total-AQP2, pS256-AQP2, and pS261-AQP2 after K^+^-depletion for 2 weeks. Under basal conditions, immunohistochemical labeling revealed that total AQP2 was strongly labeled in the apical and partially in the subapical domains of the IMCD cells in both *Atg7*^f/f^ (Fig. 3a1) and *Atg7*^Δpc^ (Fig. 3a3) mice. After K^+^-depletion, the total-AQP2 redistributed mainly into the intracellular vesicles in *Atg7*^f/f^ mice (Fig. 3a2) but redistributed diffusely throughout the cytoplasm in *Atg7*^Δpc^ mice (Fig. 3a4). On a normal diet, pS261-AQP2 was mainly labeled in the apical and partially in the subapical domains of the IMCD cells in both *Atg7*^f/f^ (Fig. 3c1) and *Atg7*^Δpc^ (Fig. 3c3) mice. After K^+^-depletion, pS261-AQP2 was located restrictively in the intracellular vesicles in *Atg7*^f/f^ mice (Fig. 3c2) but diffusely throughout the cytoplasm in *Atg7*^Δpc^ mice (Fig. 3c4). On a normal diet, pS256-AQP2 was strongly labeled on the apical membrane of the IMCD cells in both *Atg7*^f/f^ (Fig. 3e1) and *Atg7*^Δpc^ (Fig. 3e3) mice. After K^+^-depletion, on the other hand, the immunoreactivity of pS256-AQP2 in the apical membrane was markedly decreased in both *Atg7*^f/f^ (Fig. 3e2) and *Atg7*^Δpc^ (Fig. 3e4) mice. Western blot analyses for lysates of the renal inner medulla revealed that the protein expression of pS261- and pS256-AQP2 was significantly decreased after K^+^-depletion compared with controls in both *Atg7*^f/f^ and *Atg7*^Δpc^ mice (Fig. 3d,f). Densitometric quantitation revealed a decrease in expression of pS261- and pS256-AQP2 in K^+^-depleted *Atg7*^f/f^ mice to 17.63 ± 2.99% and 46.03 ± 17.24% of control levels, respectively. Furthermore, in *Atg7*^Δpc^ mice fed normal diet, the protein expression of total- and pS261-AQP2 was slightly increased to 111.07 ± 50.74% and 109.30 ± 2.38% of control *Atg7*^f/f^ mice, respectively. However, pS256-AQP2 was decreased to 70.45 ± 8.62% of control *Atg7*^f/f^ mice. In K^+^-depleted *Atg7*^Δpc^ mice, the rates of protein level decrease of total- and pS261-AQP2 were reduced compared to those of K^+^-depleted *Atg7*^f/f^ mice. In comparison, the decreased rate of pS256-AQP2 in K^+^-depleted *Atg7*^Δpc^ mice was significantly pronounced compared to that of K^+^-depleted *Atg7*^f/f^ mice. Taken together, these findings indicated that the down-regulation of AQP2 in hypokalemia was induced by a reduction of protein level, by redistribution of pS261-AQP2 into the intracellular vesicles, and by a reduction of apical plasma labeling of pS256-AQP2. In K^+^-depleted *Atg7*^Δpc^ mice, internalization of pS261-AQP2 was blocked and a pronounced reduction in the rate of apical plasma labeling of pS256-AQP2 in the IMCD cells was observed.

### pS261-AQP2 colocalizes with canonical autophagic vacuoles but not with non-canonical autophagic vacuoles

**Figure 4:**
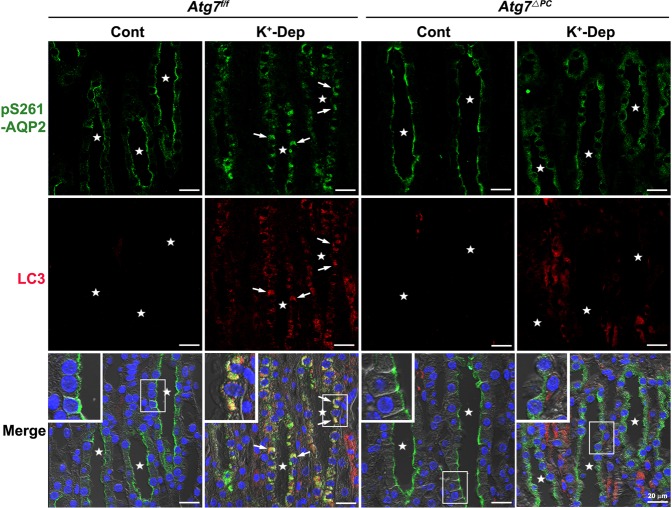
Confocal micrographs of the inner medulla of control (Cont) and K^+^-depleted (K^+^-Dep) *Atg7*^f/f^ and *Atg7*^Δpc^ mouse kidneys illustrating double labeling for pS261-AQP2 (green) and LC3 (red). Insets are higher magnification of the areas indicated by the rectangles. In K^+^-Dep *Atg7*^f/f^ mice, pS261-AQP2-positive intracellular vesicles are colocalized with LC3 (arrows). In contrast, in K^+^-Dep of *Atg7*^Δpc^, pS261-AQP2 is diffusely expressed in the cytoplasm of inner medullary collecting duct (IMCD) cells and is not colocalized with LC3. Stars indicate the lumen of inner medullary collecting ducts.

**Figure 5:**
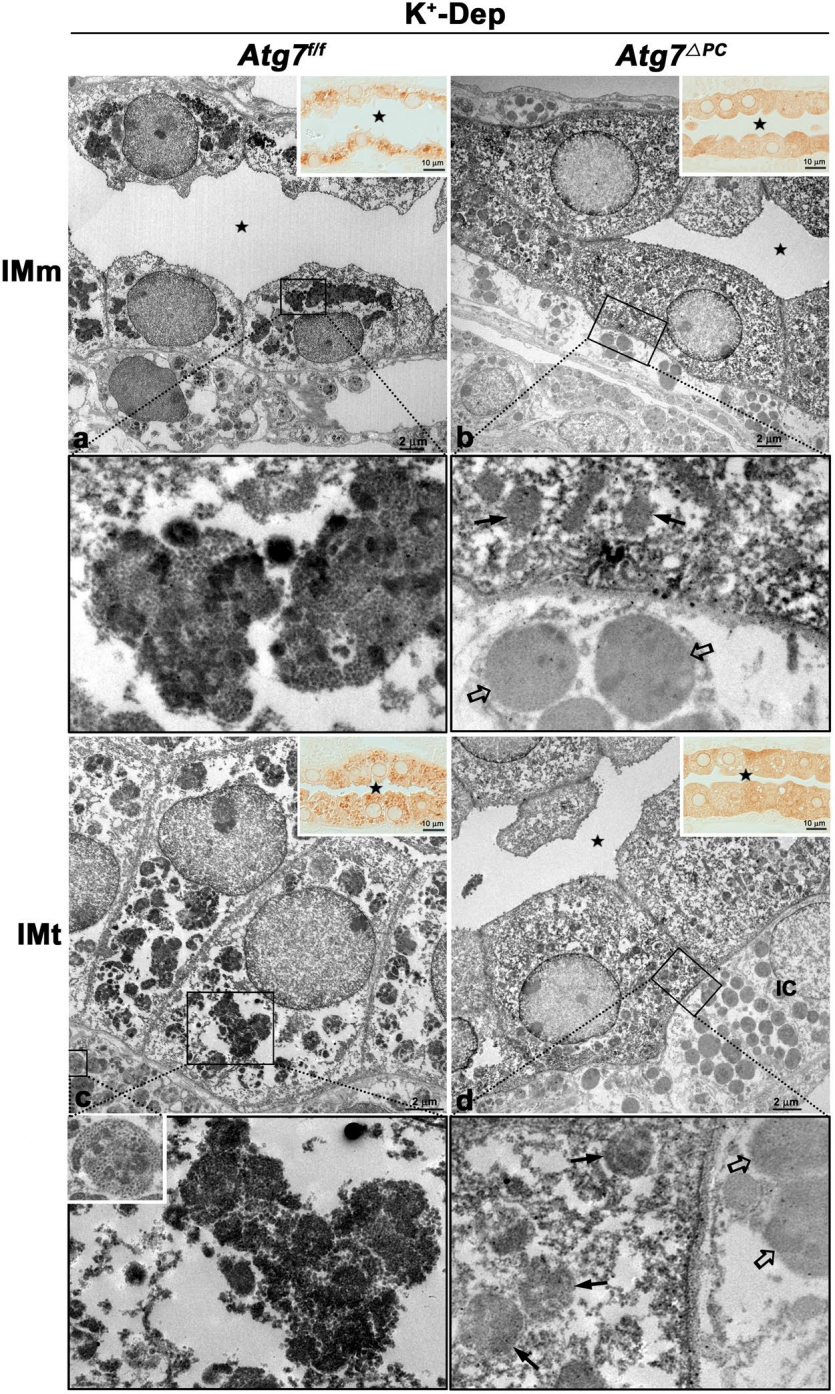
Immunoelectron micrographs of the middle (IMm) (**a**,**b**) and terminal (IMt) (**c**,**d**) parts of the inner medulla of K^+^-depleted (K^+^-Dep) *Atg7*^f/f^ (**a**,**c**) and *Atg7*^Δpc^ (**b**,**d**) mice illustrating immunostaining for pS261-AQP2. Inserts are light micrographs of 1 μm-thick semi-thin sections of the same group. Lower panels are higher magnification of the areas indicated by the rectangles in upper panels. pS261-AQP2 is intensely labeled in the large and irregular shaped canonical autophagosomes in K^+^-Dep *Atg7*^f/f^ mice (**a**,**c**), but not labeled in the small and regular shaped non-canonical autolysosomes (arrows) in K^+^-Dep *Atg7*^Δpc^ mice (**b**,**d**). Note that pS261-AQP2 is diffusely expressed throughout the cytoplasm in the inner medullary collecting duct cells (**b**,**d**). Open arrows indicate the pS261-AQP2-negative autophagic vacuoles in the interstitial cells (IC). Stars indicate the lumen of inner medullary collecting ducts (**a**–**d**).

**Figure 6:**
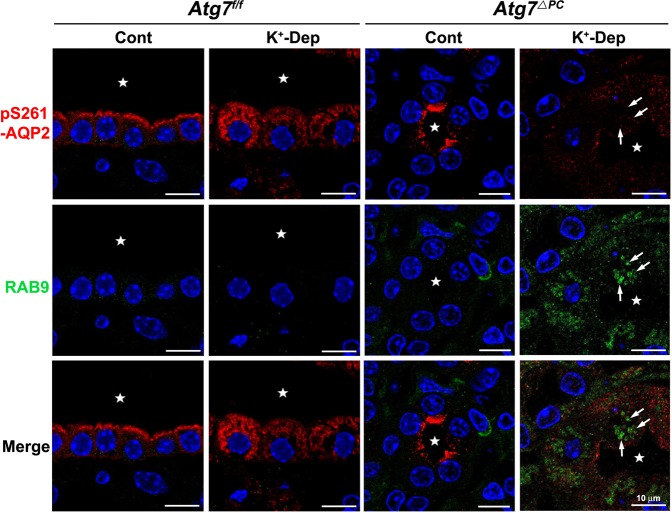
Confocal micrographs of the inner medulla of control (Cont) and K^+^-depleted (K^+^-Dep) *Atg7*^f/f^ and *Atg7*^Δpc^ mouse kidneys illustrating double labeling with pS261-AQP2 (red) and RAB9 (green). RAB9-positive puncta (green) is increased in both control and K^+^-Dep *Atg7*^Δpc^ mice; subsequently these changes in the K^+^-Dep *Atg7*^Δpc^ mice pronounced compared with *Atg7*^f/f^ mice. Note that pS261-AQP2 is not colocalized with RAB9-positive puncta (arrows) in the inner medullary collecting duct cells of K^+^-depleted *Atg7*^Δpc^ mice. Stars indicate the lumen of inner medullary collecting ducts.

Our previous findings demonstrated that hypokalemia induced autophagy and the redistribution of pS261-AQP2 from the apical or subapical domains to intracellular vesicles. Considering that cytoplasmic component and organelles are degraded by an autophagy pathway, we hypothesized that autophagy regulates water homeostasis through the degradation of pS261-AQP2. To examine this hypothesis, we performed double or multiple immunofluorescence staining using antibodies for pS261-AQP2, LC3, and RAB9, and ultrastructural immunocytochemistry for pS261-AQP2 or pS256-AQP2. Double immunofluorescence staining for pS261-AQP2 and LC3 revealed that pS261-AQP2 was colocalized with LC3-positive puncta in K^+^-depleted *Atg7*^f/f^ mice (Fig. 4). To confirm these findings at the ultrastructural level, immunocytochemical electron microscopy was used to show that the large irregular shaped autophagic vacuoles in the IMCD cells from K^+^-depleted *Atg7*^f/f^ mice were labeled with pS261-AQP2 (Fig. 5a–c). As mentioned above and reported elsewhere^36,37^, non-canonical autophagy was induced by Atg7 knockout especially under conditions of K^+^-depletion. However, in K^+^-depleted *Atg7*^Δpc^ mice, no immunolabeling for pS261-AQP2 was detected in the small round autophagic vacuoles (Fig. 5b–d). As expected based on the light microscopic immunohistochemical results (Fig. 3e), we confirmed that no immunoreactivity for pS256-AQP2 was observed in the autophagic vacuoles of the IMCD not only in K^+^-depleted *Atg7*^f/f^ mice but also in K^+^-depleted *Atg7*^Δpc^ mice (Supplementary Fig. S5). Subsequently, to confirm whether the degradation of pS261-AQP2 in K^+^-depleted *Atg7*^Δpc^ mice occurred also through the non-canonical autophagic pathway, we performed double immunolabeling for pS261-AQP2 and RAB9, which is essential for membrane expansion and fusion in alternative autophagy. We found that pS261-AQP2 was not colocalized with RAB9-positive puncta in the IMCD cells of K^+^-depleted *Atg7*^Δpc^ mice (Fig. 6). Rather, pS261-AQP2 demonstrates a similar redistribution pattern as total AQP2, suggesting that pS261-AQP2 is subject to the same degradation pathway as total AQP2 as a consequence of hypokalemia. These findings indicate that the degradation of pS261-AQP2 after K^+^ depletion is mediated by LC3-positive/RAB9-negative canonical autophagy, but not by LC3-negative/RAB9-positive non-canonical autophagy.

### pS261-AQP2 does not colocalize with SQSTM1

**Figure 7:**
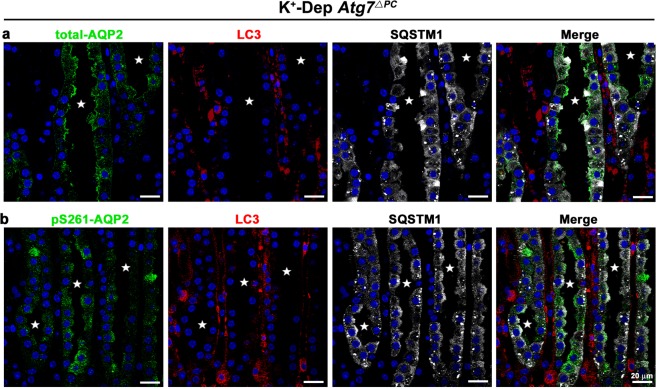
Confocal micrographs of inner medulla of K^+^-depleted (K^+^-Dep) *Atg7*^Δpc^ mice illustrating triple labeling with total-AQP2 (green, **a**) or pS261-AQP2 (green, **b**), LC3 (red), and SQSTM1 (white). In K^+^-Dep *Atg7*^Δpc^ mice, total-AQP2 or pS261-AQP2 do not colocalized not only with LC3, but also with SQSTM1. Stars indicate the lumen of inner medullary collecting ducts.

As shown in Fig. 1c and d, SQSTM1 was significantly increased in the IMCD cells after *Atg7* deletion. Substantial evidence has been reported that both ubiquitinated and non-ubiquitinated proteins are aggregated by SQSTM1, which recruits a phagophore through direct interaction with the LC3 autophagic adaptor, and are continuously regulated by autophagic clearance^42,43^. To identify whether SQSTM1 is involved in degradation of AQP2, we performed multiple immunostaining for SQSTM1, LC3, and total AQP2 or pS261-AQP2. SQSTM1 did not colocalize either with total-AQP2, pS261-AQP2, or with LC3 in K^+^-depleted *Atg7*^Δpc^ mice (Fig. 7). These findings indicate that SQSTM1 is not involved in the degradation of total- and pS261-AQP2.

### AQP2 is increased in urine after Atg7 deletion

As the amount of total- and pS261-AQP2 was decreased in K^+^-depleted *Atg7*^f/f^ and, to a greater degree, in *Atg7*^Δpc^ mice, we postulated that AQP2 might be excreted into the urinary space by exosome secretion in hypokalemia. To test this hypothesis, we examine the urinary exosome secretion of total-AQP2. As shown in Supplementary Fig. S6, the total urinary- AQP2 excretion was significantly increased in K^+^-depleted *Atg7*^f/f^ mice and these changes were markedly pronounced in *Atg7*^Δpc^ mice after K^+^-depletion. Similar data were obtained for CD63, which is a general marker of exosomes (Supplementary Fig. S6). These findings suggest that total-AQP2 containing vesicles are excreted into the urinary space in hypokalemia.

## Discussion

Although several studies have shown that a substantial decrease in AQP2 expression is one of the causes of NDI induced by prolonged hypokalemia^2,3^, the mechanism of down-regulation is not fully understood. Marples *et al*.^2^ reported that inclusion bodies in the collecting duct cells, which represent one of the morphologic characteristics of prolonged hypokalemia, are presumably involved in the degradation of AQP2 using an 11-day K^+^ deprivation rat model. Subsequently, Khositseth *et al*.^44^ demonstrated that an early reduction in AQP2 protein was possibly related with the activation of autophagy in hypokalemia induced by restricting dietary K^+^ for a period of 1–3 days.

In the current study, we generated a prolonged K^+^ deprivation mouse model by severely restricting dietary K^+^ for a period of 2 weeks. Our findings are consistent with previous studies demonstrating polyuria with alterations in renal concentrating ability and morphology occurring in prolonged K^+^ deprivation models^1,2,21,22,45^. We observed that cytoplasmic droplets accumulated not only in the IMCD cells but also in other cells in the renal papilla in the K^+^-restricted mice kidney, as previously reported in several studies^24,45^. In addition, the present report is the first to confirm that these cytoplasmic droplets represent LC3-positive autophagic vacuoles using light and electron microscopic immunocytochemistry. In response to hypokalemia from a K^+^-restricted diet for 2 weeks, total-AQP2 and pS261-AQP2 were redistributed from the apical or subapical domains to intracellular LC3-positive autophagic vacuoles. Furthermore, these proteins were not co-localized with RAB9, indicating that AQP2-containing vacuoles comprise canonical autophagic vacuoles.

Notably, downregulation of AQP2 by canonical autophagic degradation in hypokalemia is not the results of an unspecific downregulation of proteins. Jung *et al*.^45^ previously demonstrated that the cytoplasmic droplets induced by prolonged hypokalemia were not labeled for urea transporter-A1 using the immunogold method. Furthermore, several studies have demonstrated that the mRNA expression and protein abundance of colonic H^+^-K^+^-ATPase are rather increased in K^+^-deprived rats^46^. These results suggest that the canonical autophagic degradation of AQP2 and the suppression of AQP2 in the collecting duct is a specific response to K^+^ deprivation. Overall, these findings indicate that canonical autophagy mediates water homeostasis by regulating the degradation of total- and pS261-AQP2, underscoring the clinical importance of autophagy in maintaining water balance.

**Figure 8:**
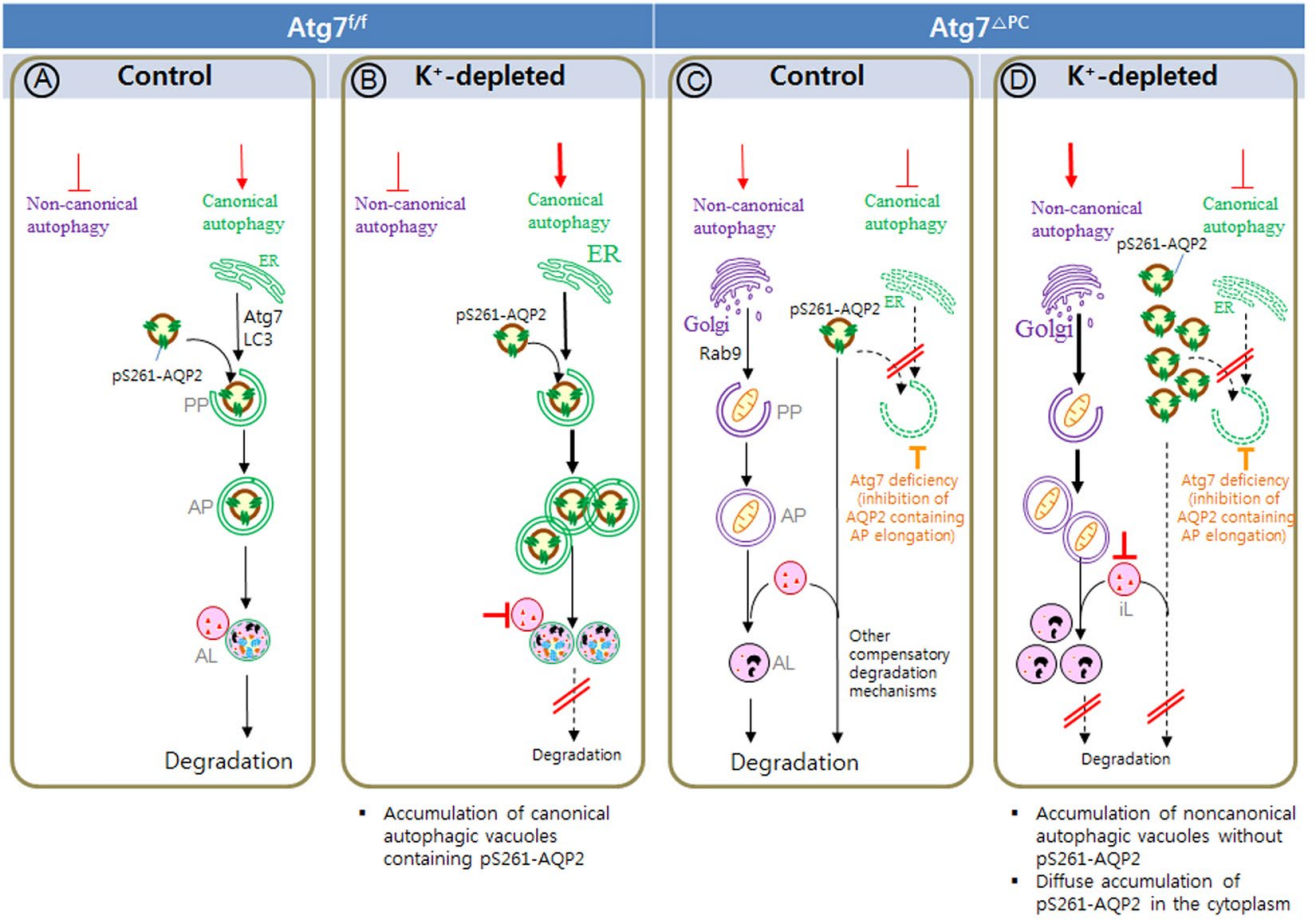
Schematic diagram summarizing the regulation of canonical autophagy and non-canonical autophagy in pS261-AQP2 degradation, and the cellular accumulation of different autophagic structures in inner medullary collecting duct (IMCD) cells of control (**a**,**c**) and K^+^-depleted (**b**,**d**) *Atg7*^f/f^ (**a**,**b**), and *Atg7*^Δpc^ (**c**,**d**) mice. Depicted are the relative amounts of phagophores (PP), autophagosomes (AP), and autolysosomes (AL). (**a**) Under normal condition of *Atg7*^f/f^ mice, pS261-AQP2 degradation is regulated by canonical autophagy. There is no accumulation of autophagic vacuoles, because of normal autophagic flux. (**b**) In K^+^-Dep *Atg7*^f/f^ mice, canonical autophagosomes containing pS261-AQP2 are accumulated in IMCD cells as a result of the blockade of autophagic flux by the inactivation of lysosome. Non canonical autophagy is not activated in both control (**a**) and K^+^-Dep (**b**) *Atg7*^f/f^ mice. (**c**) When canonical autophagy is suppressed by *Atg7* deletion, which is accompanied by enhanced activity of other lysosomal degradation mechanisms. These compensating mechanisms for canonical autophagy deficiency, resulting in no significant accumulation of AQP2 in the IMCD cells of control *Atg7*^Δpc^ mice. (**d**) In K^+^-Dep *Atg7*^Δpc^ mice, pS261-AQP2 is accumulated throughout the cytoplasm as a result of impairment of compensatory other lysosomal degradation mechanisms by inactivated lysosome. Non-canonical autophagic vacuoles without AQP2 are also accumulated as a result of impairment of non-canonical autophagy flux by inactivated lysosome.

Generally, it has been believed that *Atg5* and *Atg7* are essential for mammalian autophagy^47,48^. As we expected that, in the K^+^-depleted principal cell-specific *Atg7*-knockout mice, a marked decrease of immunoreactivity for LC3-II was observed restrictively in the IMCD cells, whereas immunoreactivity for LC3-II remained in the other structures including interstitial cells and thin limb cells of Henle’s loop. Furthermore, SQSTM1 was significantly increased only in the IMCD cells of *Atg7*^Δpc^ mice especially with K^+^-depletion for 2 weeks, suggesting that, in the renal papilla, Atg7-dependent canonical autophagy was selectively inhibited in the IMCD cells of *Atg7*^Δpc^ mice. These immunohistochemical results explained that the reason why even in the renal papilla of *Atg7*^Δpc^ mice had signals for LC3-II in the applied lysates, in contrast to the results of Komatsu *et al*.^48^ who generated the conditional knockout mice of *Atg7* mouse observed no LC3-II in ATG7-deficient tissues. Recently, in addition to canonical autophagy, Nishida *et al*.^36^ demonstrated the existence of an LC3-ATG7-independent autophagy or non-canonical autophagy^49^, which has been considered to be regulated by RAB9^36,37,50^. This is consistent our findings showing that autophagic vacuoles were observed in *Atg7*-deficient mice after K^+^ depletion, which suggested the existence of an ATG7-independent macroautophagy pathway. Notably, RAB9, which was not activated in *Atg7*^f/f^ mice in response to hypokalemia, was markedly activated in Atg7 deficient mice. These findings let us to postulate that the non-canonical autophagy pathway might serve as a compensatory mechanism in the canonical autophagy-deficient state to maintain cellular homeostasis. However, RAB9-positive autophagic vacuoles in *Atg7* deficient mice were only faintly labeled with total-AQP2 or pS261-AQP2. Furthermore, activation of the non-canonical autophagy was not able to rescue canonical autophagy deficient mice from the urinary concentration defects in hypokalemia. Our transmission electron microscopy data indicated that Atg7-deleted IMCD cells contained well developed Golgi complexes, which are essential for the formation of the autophagosomes of non-canonical autophagy^36,51^, reinforcing the concept that a non-canonical autophagy is operant in *Atg7*-deficient IMCD cells of Atg7^f/f^ mice to compensate for the functional loss of canonical autophagy. The reason of that no non-canonical autophagic vacuoles are observed under basal conditions in *Atg7*-deficient mice might be a result of normal autophagic flux by active lysosomes (Fig. 8c). In contrast, in *Atg7*-deficient mice after K^+^ depletion, LC3-negative, non-canonical autophagic vacuoles are accumulated as a result of the blockade of autophagic flux by the inactivation of lysosomes (Fig. 8d). These findings suggest that non-canonical autophagy is activated in canonical autophagy deficient mice but that this activated non-canonical autophagy has only a limited role in AQP2 degradation. Because the ATG7-dependent autophagy-impaired IMCD cells displayed minimal functional defect and did not lose their autophagy response, it might be surmised that the IMCD cells have compensated in some way to resume obligatory ATG7-dependent canonical autophagy. In *Atg7*-deficient mice after K^+^ depletion, total- and pS261-AQP2 redistribute throughout the cytoplasm from apical plasma membrane (Fig. 8d).

The impairment of the autophagic lysosomal degradation of total-AQP2 and pS261-AQP2 in hypokalemia caused a severe urinary concentration defect. Whereas the precise mechanism underlying this effect is unclear, three plausible explanations exist. The first possibility arises from the decrease of IMCD cell apical labeling by pS256-AQP2 in both *Atg7*^f/f^ and *Atg7*^Δpc^ mice in response to hypokalemia, which is pronounced in the *Atg7*^Δpc^ mice (Fig. 8b,d). The importance of pS256-AQP2 in AQP2 trafficking from the intracellular vesicles to the surface membrane has previously been suggested. Vasopressin acts on the V2 receptor, causing phosphorylation at Ser 256 and trafficking of the modified AQP2 to the surface membrane^52–54^. Eto *et al*. revealed that phosphorylation at Ser256 increased water permeability^55^. Furthermore, even under basal conditions, with free access to water, the reason why K^+^-depleted *Atg7*^Δpc^ mice had an increase in urine volume and a decrease in urine osmolarity is also because the reduction of protein level and apical labeling of pS256-AQP2. Therefore, impairment of the apical labeling by pS256-AQP2 in autophagy-deficient mice likely caused the urinary concentration defect.

The second possible mechanism underlying this defect is exosomal secretion of AQP2. Secretion of intact AQP2 into the urine was first identified by Kanno *et al*.^56^ Subsequently, the mechanism of AQP2 delivery into the urine was identified to occur via exosome secretion^57–59^. Under a steady-state condition, an increase in urinary AQP2 excretion might reflect either an increase in synthesis or a decrease in degradation. As shown in Sup Fig S6, Atg7 deficiency resulted in a failure in the generation of canonical autophagosomes and thereby, a large fraction of AQP2-containing vesicles were delivered from the intracellular compartment to the lumen by exosome secretion. Increased exosome secretion of AQP2 is accompanied by a decrease in renal medullary AQP2 protein as has been previously reported by Higashijima *et al*.^60^, which might therefore cause the observed urinary concentration defect.

The third potential mechanism underlying the urinary concentration defect is SQSTM1 mediated degradation of AQP2. Substantial evidence supports that the autophagic adaptor SQSTM1 acts as a cargo receptor for the degradation of ubiquitinated substrates^61^. For example, ubiquitinated proteins are aggregated by SQSTM1, which recruits a phagophore through direct interaction with LC3. The cellular contents in SQSTM1 are continuously regulated by autophagic clearance^42,62,63^. It has recently been demonstrated that SQSTM1 also has the ability to target non-ubiquitinated proteins for degradation^43^. In this study, we confirmed that the accumulation of SQSTM1 in the cytoplasm of IMCD cells occurred after Atg7 deletion, as previously reported^64,65^. However, we further observed that SQSTM1 colocalized neither with total-AQP2 nor with pS261-AQP2 in K^+^-depleted *Atg7*^Δpc^ mice, suggesting that SQSTM1 was not involved in the degradation of total- and pS261-AQP2.

In summary, our results demonstrated that urinary concentrating defect in hypokalemia is induced by reduced AQP2 phosphorylation, resulting in a reduction of apical labeling of pS256-AQP2. Our data demonstrated, also, that autophagy, occurring primarily through a LC3/Atg7-dependent canonical autophagy pathway, is involved in the degradation of total- and pS261-AQP2 in IMCD cells. These findings provide new insights into the mechanisms of AQP2 degradation and water homeostasis.

## Materials and Methods

### Generation of principal cell-specific *Atg7* knockout mice and K^+^ depletion model

To generate mice with an *Atg7* deletion specifically in the principal cells of the collecting duct (*Atg7*^Δpc^), we crossed *Atg7*^*flox/flox*^ mice (Atg7^f/f^) (kindly provided by Dr. Komatsu, School of Medicine, Niigata University) with aquaporin 2-cre mice (Stock No. 006881, The Jackson Laboratory). The genotypes of offspring were determined by polymerase chain reaction (PCR) analysis using genomic DNA obtained from tails of mice and transgene-specific primers. All mouse lines were bred onto a C57BL/6 background. All animal experiments were approved by the Ethics Committee of Bucheon St. Mary’s Hospital in accordance with the institutional guidelines and regulations on the use of laboratory animals.

In this study, we used only adult male mice (20–25 g, 8 weeks old). The mice were divided into four groups (n = 8–10/group): *Atg7*^*f/f*^ control and K^+^-depletion, *Atg7*^Δpc^ control and K^+^-depletion. The control group was fed normal diet (K^+^; 25 gm, Research Diet Inc.) and distilled water and the K^+^-depletion group was fed K^+^-free diet (K^+^; 0 gm, Research Diet Inc.) and distilled water for 2 weeks.

To monitor autophagy in control and K^+^-depleted *Atg7*^*f/f*^ mice, we introduced GFP-LC3 as a marker for autophagy used green fluorescent protein (GFP)-LC3 transgenic mice (Stock No. RBRC00806, Riken BioResource Center, Japan).

Two weeks later, mice were treated in metabolic cage for collecting 24 hours urine and then anesthetized and blood was collected from abdominal aorta. Blood analysis was performed by i-STAT system with CHEM8+ cartridge (Abott Inc.) Urine analysis was carried out by Samgwang Medical Foundation (Hitachi 7600-110, Urisys 2400, etc.).

### Antibodies

The antibodies used included those against LC3B for western blotting (Cat. No. L7543, Sigma-Aldrich Inc.) and for immunohistochemistry (Cat. No. 0231-100/LC3-5F10, Nanotools), SQSTM1 (Cat. No. GP62-C, Progen), RAB9 (Cat. No. ab2810, Abcam), pS261-AQP2 (Cat. No. ab72383, Abcam), pS256-AQP2 (Cat. No. ab109926, Abcam & kindly provided by Prof. Tae-Hwan Kwon, Kyungpook National University, Korea), and total AQP2 (Cat No. AQP-002, Alomone).

### Western blotting

After experimental treatment, the mice were anesthetized and perfused with phosphate buffered saline (PBS, pH 7.4). The inner medulla of the kidney was isolated and homogenized with boiling lysis buffer (1.0% sodium dodecyl sulfate (SDS), 1.0 mM sodium orthovanadate, and 10 mM Tris-Cl, pH7.4) as previously described^66^ and protein concentration was determined using the BCA kit (Cat. No. 23225, Pierce Biotechnology Inc.). Equal amounts of the protein were separated by SDS-polyacrylamide gel electrophoresis, and transferred onto nitrocellulose membranes. Membranes were blocked with PBS containing 0.1% Tween-20 and 5% skim milk and then incubated with primary antibodies overnight. The next day, the membranes were washed and incubated with the appropriate secondary antibodies and the signals were detected using a western blotting luminol reagent kit (Cat. No. sc 2048, Santa Cruz Biotechnology) and quantified by densitometry with a Multi Gauge instrument (v 3.0, Fusifilm). Quantification the immunoblot signals of three independent experiments performed in triplicate. The signals were scanned, and the amounts of target proteins were quantified in arbitrary unit ± SE. The precise methods were described in a previous report^67^.

### Immunohistochemistry for light microscopy

After experimental treatment, the mice were anesthetized, perfused with PBS, and then fixed with a 2% paraformaldehyde-lysine-periodate solution for 10 min. The kidneys were removed and cut into 1–2 mm thick slices, which were postfixed by immersion in the same fixative overnight at 4 °C. The kidneys were then embedded in poly (ethylene glycol) (400) distearate (Cat. No. 01048, Polysciences Inc.) and cut transversely at a thickness of 4 μm using a microtome. Tissue sections were hydrated with graded ethanol and rinsed in tap water. The sections were then treated with a retrieval solution (pH 6.0), methanol containing 5% H_2_O_2_, 0.5% Triton X-100 in PBS, normal donkey serum, and subsequently incubated with primary antibodies overnight at 4 °C. The next day, after washing in PBS, the tissue sections were incubated with the appropriate secondary antibodies and the signals were visualized using a 0.05% DAB and 0.01% H_2_O_2_ mixture. Then sections were next washed with distilled water, dehydrated with graded ethanol and xylene, mounted in Canada balsam, and examined by light microscopy using Olympus BX51. The precise methods were described in a previous report^68^.

### Immunofluorescence analysis

Tissue sections were hydrated with graded ethanol and rinsed in tap water. The sections were then treated with a retrieval solution (pH 6.0), 0.5% Triton X-100 in PBS, normal donkey serum, and subsequently incubated with primary antibodies overnight at 4 °C. The next day, after washing in PBS, the tissue sections were incubated with the fluorescence-labeled appropriate secondary antibodies and mounted in Vectashield mounting medium (Vector Laboratories). Images were acquired using a Zeiss LSM 700 Confocal microscope (Carl Zeiss).

### Transmission electron microscopy

The mice were anesthetized, perfused with PBS, and then fixed with a 2% paraformaldehyde-2.5% glutaraldehyde solution for 10 min. The inner medulla of the kidneys were removed and cut into 0.5–1 mm pieces and postfixed by immersion in the same fixative overnight at 4 °C. The tissues were then dehydrated with graded ethanol and embedded in Poly/Bed 812 resin (Polyscience). Ultrathin sections were cut and photographed using a JEOL JEM-1010 transmission electron microscope.

### Immunocytochemistry for electron microscopy

After fixation, the kidney sections were cut transversely using a vibratome to a thickness of 50 μm and processed for immunocytochemistry. Sections were washed with PBS and treated with 50 mM NH_4_Cl. Prior to incubation with the primary antibodies, the sections were pretreated with a graded series of ethanol and then incubated for 4 h in PBS containing 1% bovine serum albumin (BSA), 0.05% saponin, and 0.2% gelatin. The tissue sections were next incubated with primary antibodies overnight at 4 °C. After several washes in PBS containing 0.1% BSA, 0.05% saponin, and 0.2% gelatin, the sections were incubated with secondary antibodies, washed, and colorized with a 0.1% DAB and 0.01% H_2_O_2_ mixture for 10 min. The tissue sections were treated with 1% glutaraldehyde and 1% OsO_4_, dehydrated with graded ethanol, and embedded with Poly/Bed 812 between polyethylene vinyl sheets. Sections from the inner medulla were excised and glued onto empty blocks of Poly/Bed 812. Ultrathin sections were cut and photographed using a JEOL JEM-1010 transmission electron microscope. The precise method was as described in a previous report^69^.

### Quantification of the autophagic area

The autophagic area was measured using the Multi Gauge system. IMCD cells were chosen and the autophagic area (autophagic area (%) = total autophagic vacuoles area/cytoplasm area (whole cell area–nucleus area) was measured. In this study, over 20 images per group were measured.

### Enzyme-linked immunosorbent assay

Fifty microliter of urine samples or standard AQP2 synthetic peptide corresponding to amino acis 257 to 271 of human AQP2 were added to coat a Maxisorp 96-well microplate (NUNC, Rochester, NY, USA). After incubation overnight at 4 °C, the plate was washed, and blocked with 3% bovine serum albumin (BSA)/phosphate-buffered saline (PBS). Then the blocking solution was removed, and the diluted rabbit polyclonal antibody AQP2 was added to each well. After incubation at 37 °C for two hours, the plate was washed, and diluted horseradish peroxidase (HRP)-conjugated anti-rabbit immunoglobulin G (IgG) (Jackson ImmunoResearch Laboratories, West Grove, PA, USA) was added to each well. After incubation at 37 °C for one hour, the plate was washed, and 150 $\mu \ell$ of the substrate solution [2, 2′-azino-bis (3-ethylbenzthiazoline-6-sulfonic acid)] (Sigma, St. Louis, MO, USA) was added. After incubation at room temperature for 30 minutes in the dark, the reaction was stopped by adding 150 $\mu \ell$ of 1% SDS, and the absorbance at 405 nm was measured.

### Statistical analyses

All data are presented as the means ± SD. Differences between groups were evaluated using the Student’s t-test or one-way analysis of variance. Statistical significance was determined as *P* < 0.05.

## Supplementary information

Supplementary Information
