# Supplementary material for: Atg7-dependent canonical autophagy regulates the degradation of aquaporin 2 in prolonged hypokalemia
Source: Sci Rep. 2019 Feb 28;9:3021. doi: 10.1038/s41598-019-39702-4 (PMC6395725; doi:10.1038/s41598-019-39702-4)
Supplement: Supplementary file 1 — Supplementary Information [file 41598_2019_39702_MOESM1_ESM.pdf]

***Atg7*-dependent canonical autophagy regulates the degradation of aquaporin 2 in prolonged hypokalemia**

Wan-Young Kim[1], Sun Ah Nam[1], Arum Choi[1], Yu-Mi Kim[1], Sang Hee Park[2], Hong Lim Kim[3], Hyang Kim[4], Ki-Hwan Han[5], Chul Woo Yang[6], Myung-Shik Lee[7], Yong Kyun Kim[1,6] & Jin Kim[1]

[1]Department of Anatomy and Cell Death Disease Research Center, College of Medicine, The Catholic University of Korea, Seoul, Korea. [2]Institute of Clinical Medicine Research of Bucheon St. Mary's Hospital, Bucheon, Korea. [3]Integrative Research Support Center, College of Medicine, The Catholic University of Korea, Seoul, Korea. [4]Division of Nephrology, Kangbuk Samsung Hospital, Sungkyunkwan University, School of Medicine, Seoul, Korea. [5]Department of Anatomy, Ewha Womans University School of Medicine, Seoul, Korea. [6]Department of Internal Medicine, College of Medicine, The Catholic University of Korea, Seoul, Korea. [7]Severance Biomedical Science Institute, College of Medicine, Yonsei University, Seoul, Korea.

Correspondence: Jin Kim, M.D., Ph.D.

Address: Department of Anatomy and Cell Death Disease Research Center, College of Medicine, The Catholic University of Korea, Seoul, Korea, 222 Banpo-Daero, Seocho-Ku, Seoul, 06591, Korea

Telephone: 82-2-2258-7258

Fax: 82-2-536-3110

E mail: jinkim@catholic.ac.kr

Correspondence: Yong Kyun Kim, M.D., Ph.D.

Address: Department of Internal Medicine, Bucheon St. Mary's Hospital, College of Medicine, The Catholic University of Korea, 327 Sosa-ro, Bucheon, Gyeonggi-do, 14647 Korea

Telephone: 82-32-340-7019

Fax: 82-32-340-2667

E mail: drkimyk@catholic.ac.kr

Supplementary Information

Supplementary Table S1. Effect of AQP2-specific deficiency of Atg7 on kidney weight and biochemical parameters.

		Atg7$^{f/f}$		*Atg7*$^{\Delta PC}$	
		Control	K^+-depleted	Control	K^+-depleted
Weight	Body weight (g)	22.5 ± 0.5	19.5 ± 1.26**	24.5 ± 1.63**	23.3 ± 1.31^{+}
	Kidney weight (g)	0.16 ± 0.01	0.15 ± 0.02	0.17 ± 0.02	0.19 ± 0.02$^{**/++}$
	KW/BW (%)	100 ± 8.43	115.15 ± 10.10*	108.65 ± 1.38	123.88 ± 8.05$^{*/\#}$
Blood	Plasma Na^+ (mmol/L)	150.2 ± 1.8	147.4 ± 2.1	150.2 ± 2.0	153.2 ± 2.1$^{*/+/\#}$
	Plasma K^+ (mmol/L)	3.89 ± 0.32	2.97± 0.26**	4.27 ± 0.30	2.28 ± 0.25$^{**/++/\#\#}$
	Plasma Cl^- (mmol/L)	112.8 ± 2.4	113.3 ± 1.5	114.2 ± 3.8	119.5 ± 4.1$^{*/+/\#}$
	BUN (mg/dL)	27.2 ± 5.9	30.8 ± 4.49	27.0 ± 2.65	47.00 ± 7.79$^{*/+/\#}$
Urine	Urine volume (ml)	0.56 ± 0.37	2.48 ± 1.36**	2.37 ± 0.67**	3.02 ± 0.81$^{**/+/\#}$
	Osmolality (mosmol/kgH_2O)	2244.1 ± 421	1168.1 ± 418**	1525.7 ± 401**	974 ± 185$^{**/+/\#\#}$
	Urine Na^+ (mmol/L)	70.7 ± 18.0	38.1 ± 16.8*	52.1 ± 20.0*	26.9 ± 6.2$^{*/+/\#}$
	Urine K^+ (mmol/L)	137.6 ± 16.3	13.7 ± 6.0**	105.2 ± 32.2**	15.3 ± 3.7$^{**/\#\#}$
	Urine Cl^- (mmol/L)	132.1 ± 31.1	61.3 ± 24.9*	95.9 ± 33.2*	67.9 ± 18.0$^{*/+/\#}$

t-test vs. *Atg7*$^{f/f}$ Control * $P < 0.05$, ** $P < 0.001$

t-test vs. *Atg7*$^{f/f}$ K^+-depleted + $P < 0.05$, ++ $P < 0.001$

t-test vs. *Atg7*$^{\Delta PC}$ Control # $P < 0.05$, ## $P < 0.001$

All values are the means ± SEM.

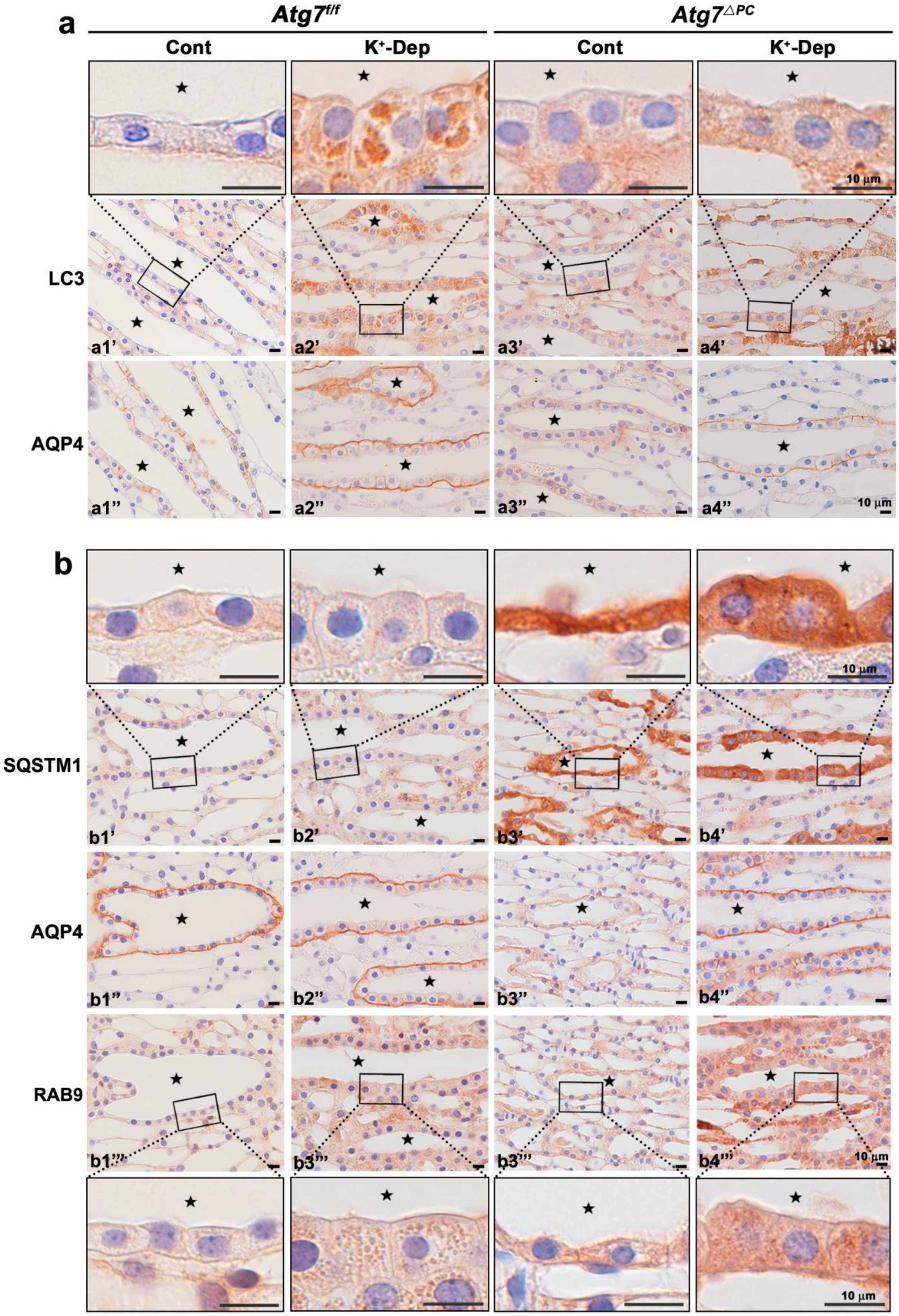

a
Atg7f/f
Atg7△PC
Cont
K+-Dep
Cont
K+-Dep
10 μm
LC3
a1'
a2'
a3'
a4'
AQP4
a1''
a2''
a3''
a4''
10 μm
b
10 μm
SQSTM1
b1'
b2'
b3'
b4'
AQP4
b1''
b2''
b3''
b4''
RAB9
b1'''
b3'''
b3'''
b4'''
10 μm
10 μm

Figure S1. Light micrographs of the inner medulla of control (Cont) and K^+-depleted (K^+-Dep) *Atg7*$^{f/f}$ and *Atg7*$^{\triangle pc}$ mouse kidneys illustrating immunolabeling of LC3 (**a1'-a4')**, SQSTM1 (**b1'-b4')** and RAB9 (**b1'''-b4''')**. Inner medullary collecting ducts (IMCDs) (stars) were identified by immunolabeling on the basolateral plasma membrane for AQP4 using consecutive sections (**a1''-a4'')** and (**b1''-b4'')**. (**a)** In the images, it can be observed that the intense immunoreactivity for LC3 in K^+-Dep *Atg7*$^{f/f}$ mice is markedly and restrictively decreased in the AQP4-positive IMCD cells in K^+-Dep *Atg7*$^{\triangle pc}$ mice. (**b)** Strong immunoreactivity for SQSTM1 is observed restrictively in the AQP4-positive IMCD cells of *Atg7*$^{\triangle pc}$ mice. Note the prominent increase of RAB9 in K^+-Dep *Atg7*$^{\triangle pc}$ mice. Boxes in (**a**, **b)** are higher magnification of the areas indicated by the rectangles in upper or lower panels.

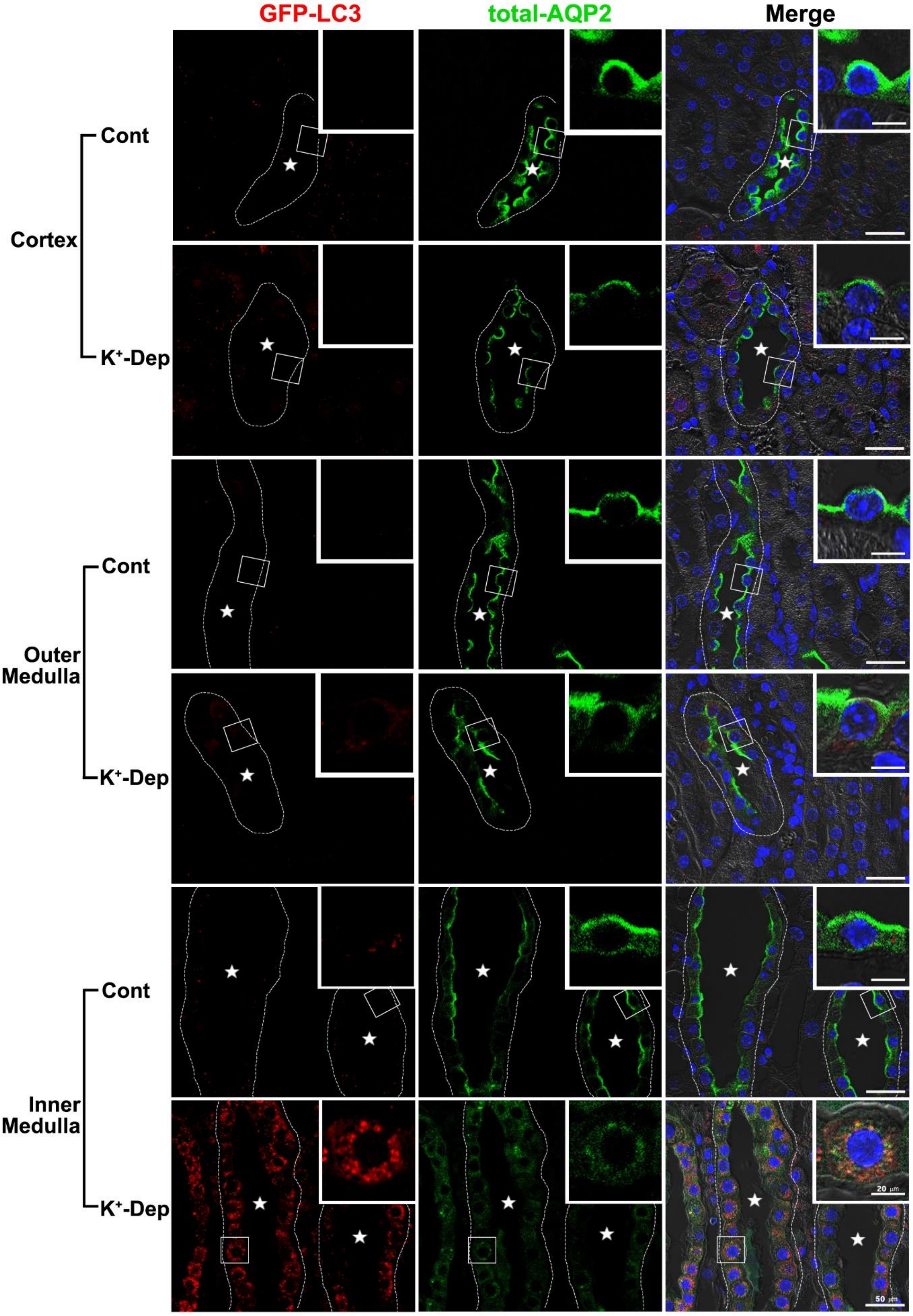

Figure S2. Confocal micrographs of the cortex, outer and inner medulla of control (Cont) and

K^+-depleted (K^+-Dep) GFP-LC3 transgenic mice illustrating double labeling with GFP-LC3 (red) and total-AQP2 (green). After potassium depletion for 2 weeks, GFP-LC3-positive dots are markedly accumulated in the inner medullary collecting duct (IMCD) cells, but a few in both cortical and outer medullary collecting duct cells. Note that total-AQP2 is colocalized with these GFP-LC3-positive puncta in the IMCD cells. Stars indicate the lumen of collecting ducts.

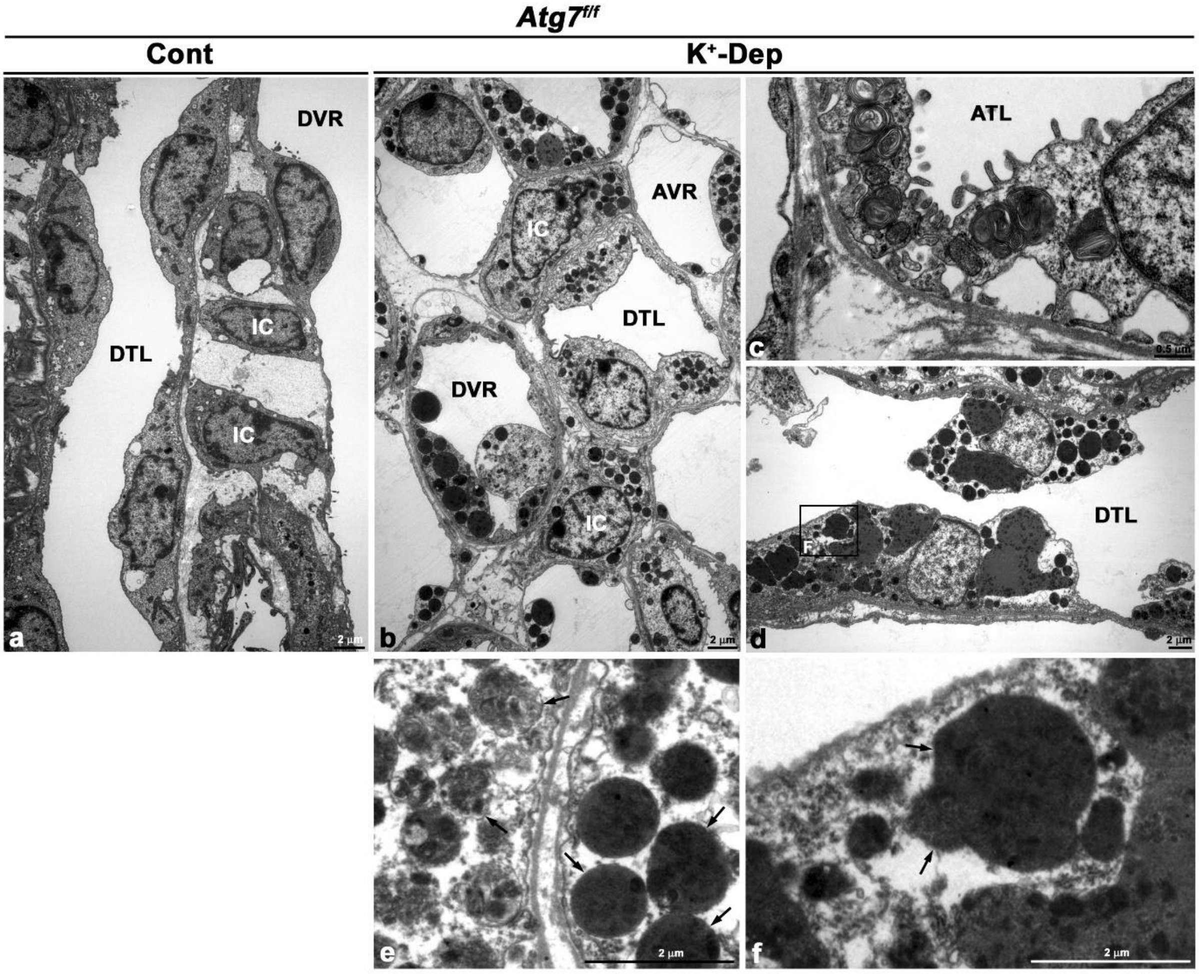

Figure S3. Transmission electron micrographs of the inner medulla of *Atg7*$^{f/f}$ mouse kidneys. (**a**). On normal diet (Cont), no autophagic vacuoles are observed in the cytoplasm of interstitial cell (IC), descending thin limb (DTL), or descending vasa recta (DVR). (**b**-**f**) After K^+-depletion (K^+-Dep), the accumulation of numerous autophagic vacuoles containing multilamellar bodies or degradation materials can be observed in the IC, DTL, ascending thin limb (ATL), DVR, and ascending vasa recta (AVR). (**f**) Higher magnifications of the areas outlined in (**d**). A single membrane (arrows) can be observed surrounding various autophagic vacuoles.

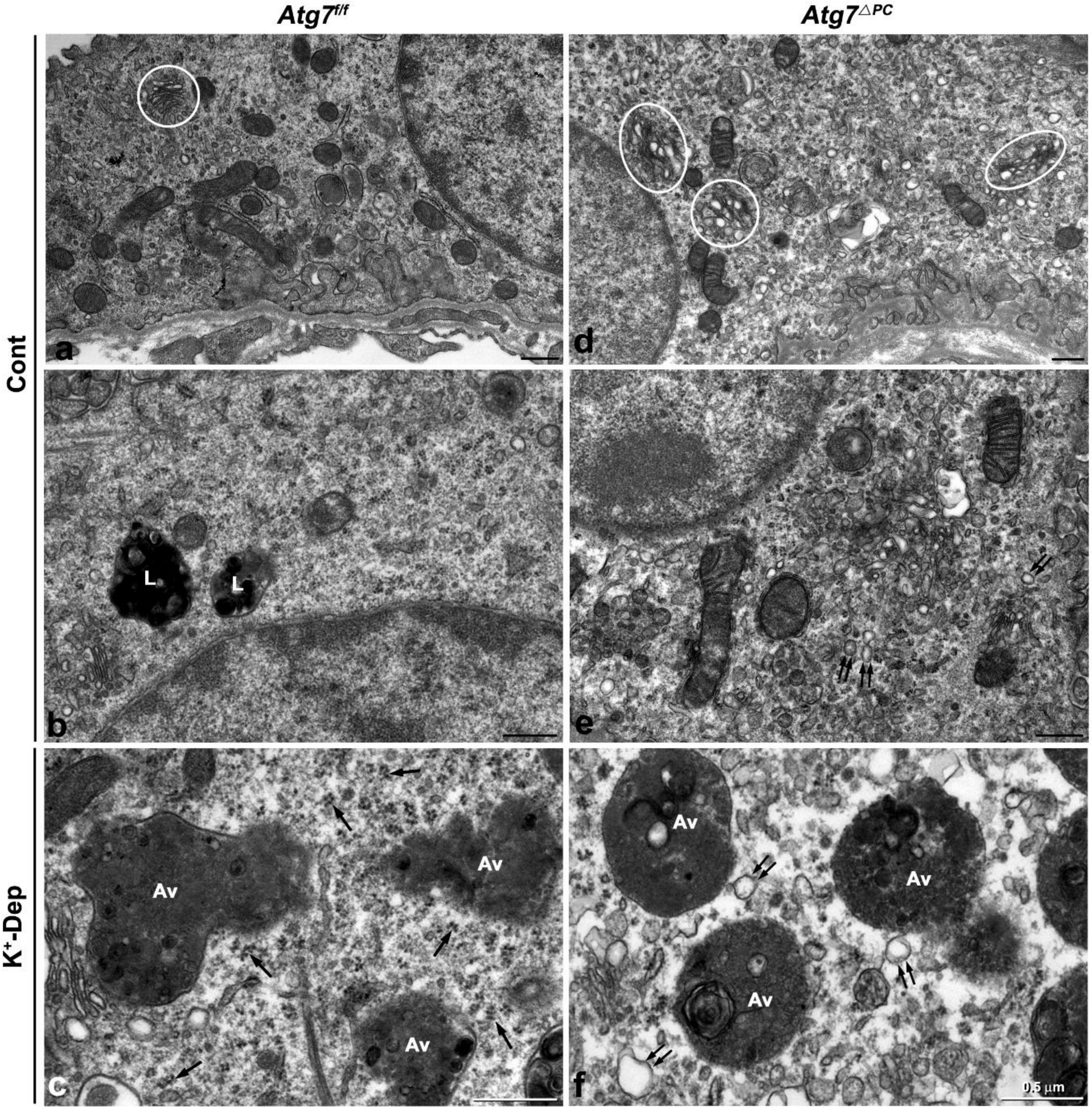

Figure S4. Transmission electron micrographs of inner medullary collecting duct (IMCD) cells. **(a-c)** In *Atg7*$^{f/f}$ mice, polysomes (arrows) are markedly increased throughout the cytoplasm in IMCD cells, as are autophagic vacuoles (Av) after K^+-depletion (K^+-Dep). **(d-f)** In *Atg7*$^{\triangle pc}$ mice, in contrast, Golgi complexes (circles) and small tubuloalveolar shaped vesicles (double arrows) are markedly increased in both control (Cont) and K^+-Dep groups.

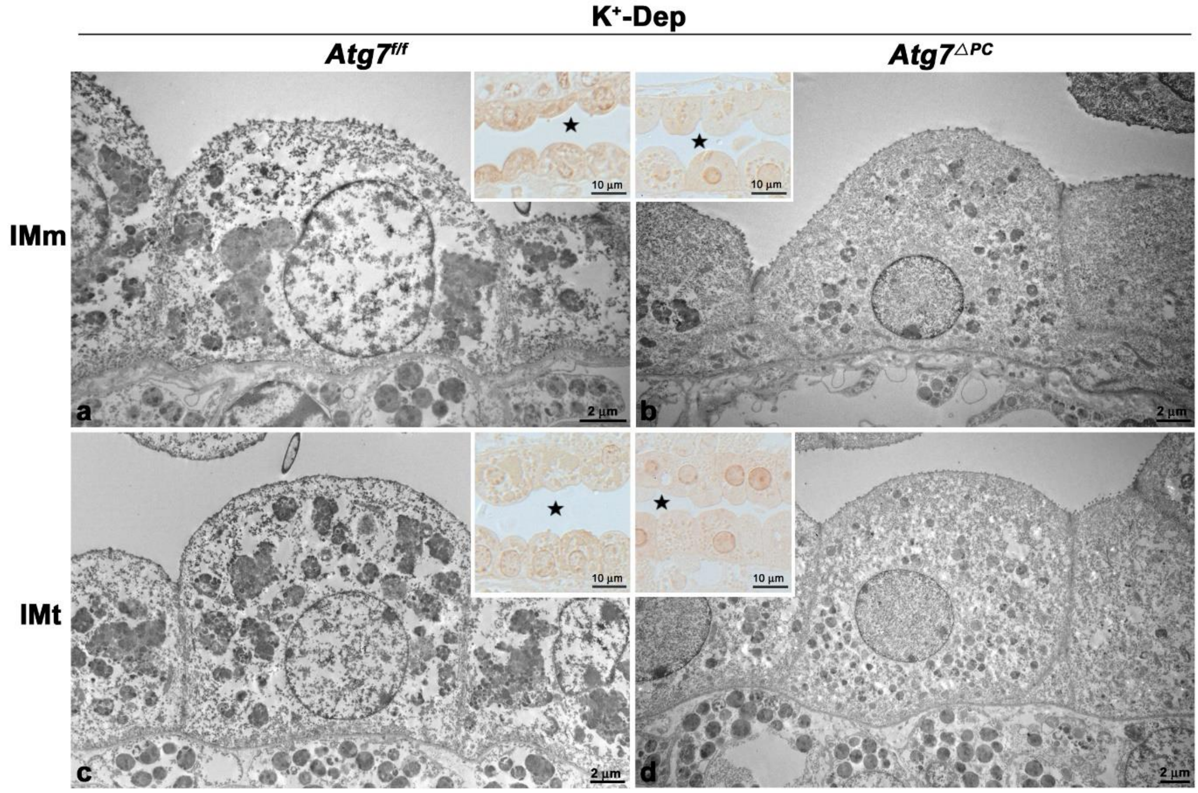

Figure S5. Immunoelectron micrographs of the middle (IMm). **(a**, **b)** and the terminal (IMt). **(c**, **d)** parts of the inner medulla of K^+ depleted (K^+-Dep) *Atg7*$^{f/f}$. **(a**, **c)** and *Atg7*$^{\triangle pc}$ **(b**, **d)** mice illustrating immunostaining for pS256-AQP2. The inserts are light micrographs of 1 m-thick semi-thin sections of the same group. No immunoreactivity of pS256-AQP2 is observed in either the canonical autophagic vacuoles in K^+-Dep *Atg7*$^{f/f}$ mice **a**, **c** or the non-canonical autophagic vacuoles in K^+-Dep *Atg7*$^{\triangle pc}$ mice **(b, d).** Stars indicate the lumen of inner medullary collecting ducts **(a-d)**.

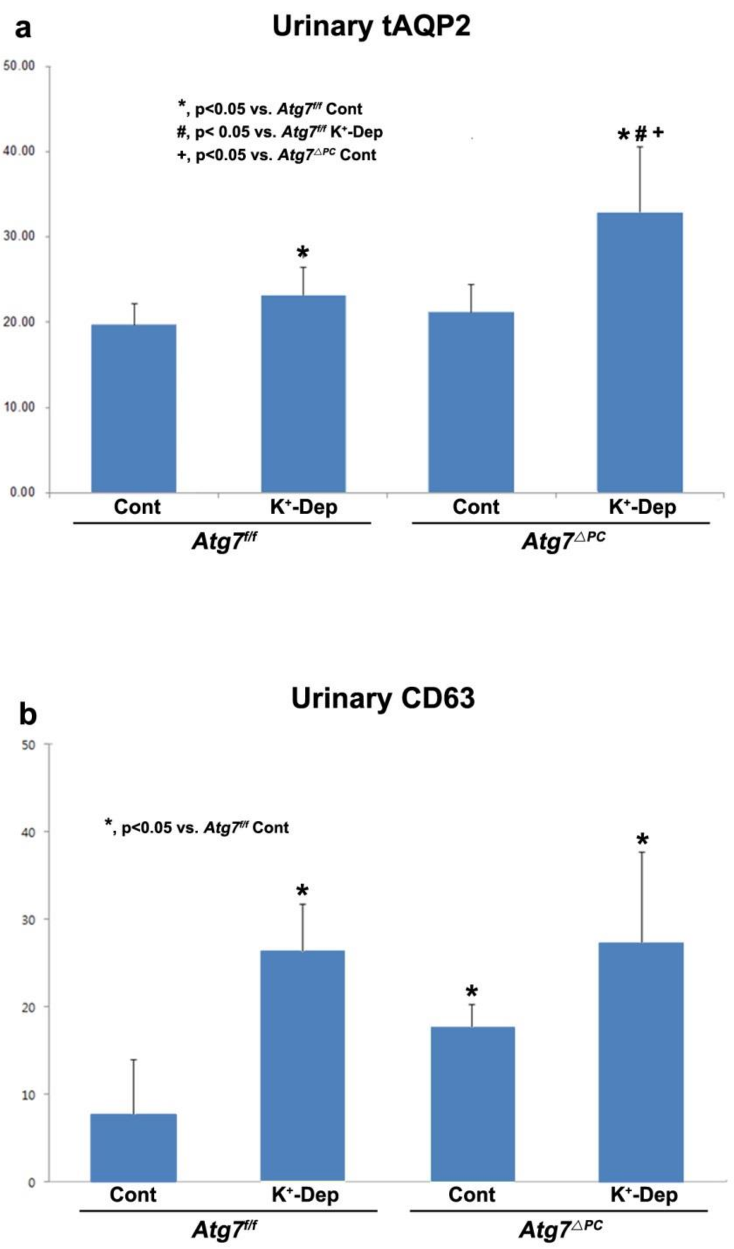

Figure S6. Enzyme-linked immunosorbent assay of urinary total-AQP2 (**a**) and CD63 (**b**). Note that urinary excretion of total-AQP2 and CD63 are significantly increased after K^+ depletion in both *Atg7*$^{f/f}$ mice and *Atg7*$^{\triangle pc}$ mice compare to those of control mice.
